# Aging With Autism Spectrum Disorder: An Exploration of Physical Activity Engagement

**DOI:** 10.1093/geroni/igaa057.1288

**Published:** 2020-12-16

**Authors:** Danielle Waldron, Beth Dugan, Jeffrey Stokes, Caitlin Coyle, John Kramer

**Affiliations:** 1 University of Massachusetts Boston, Boston, Massachusetts, United States; 2 University of Florida, Gainesville, Florida, United States

## Abstract

Adults with Autism Spectrum Disorder (ASD) participate in physical activity (PA) infrequently compared to adults in the general population. This is problematic as individuals with ASD suffer from disproportionate physical and mental health co-morbidities as well as diminished life expectancy, but do not reap the physical and mental health benefits of PA. This study used data from the National Core Indicators-In Person Consumer Survey (n=4,370; age: 18-78) to analyze factors associated with both aerobic PA and muscle strengthening (MS) activity in adults with ASD receiving state Developmental Disability Services. This research used multilevel logistic regression modeling, with mediation and moderation analyses to explore personal and environmental factors associated with PA/MS in this population. Findings indicated the following significant associations between community engagement and PA and MS: community contact (OR=1.17; p<0.001; OR=1.07; p<0.001), community group participation (OR=1.83; p<0.001; OR=1.91; p<0.001), and employment/day program participation (OR=1.32; p<0.05; OR=1.32; p<0.001). Additionally, at older ages, participants were less likely to engage in PA and MS three or more times a week (OR=0.99; p<0.05; OR=0.99; p<0.05). These findings indicate that increasing age is associated with decreased PA and MS activity in this group, while community engagement may facilitate their PA and MS activity. While much remains unknown about the population aging with ASD, it is evident that they suffer from poorer health than the general population and have experienced lifelong difficulties with socialization and communication. Greater access to community engagement opportunities may promote this population’s healthy aging, as well as support their unique social needs.

